# The ejaculate obtained on the morning of microdissection testicular sperm extraction (m-TESE) or m-TESE itself in repeated intracytoplasmic sperm injection failure due to severe male infertility? A retrospective study

**DOI:** 10.3325/cmj.2025.66.367

**Published:** 2025-10

**Authors:** Sule Atalay Mert, Mustafa Ozturk, Tugba Kinay, Ulaş Fidan, Ozhan Ozdemir, Turgay Ebiloglu, Cem Korkmaz, Seyit Temel Ceyhan

## Abstract

**Aim:**

To compare intracytoplasmic sperm injection (ICSI) outcomes between oligoasthenoteratozoospermic (OAT) men with recurrent assisted reproductive technology (ART) failures who underwent ICSI using sperm from the ejaculate on the day of a planned microdissection testicular sperm extraction (m-TESE) vs sperm obtained via m-TESE.

**Methods:**

We reviewed the outcomes of men who underwent ICSI using either ejaculate or m-TESE due to OAT and recurrent ART failure at the Department of Reproductive Endocrinology, Gülhane Training and Research Hospital, between November 2016 and January 2024. The study enrolled 172 men: 66 men in the ejaculate group and 106 in the m-TESE group. All patients had fewer sperm parameters in two subsequent semen analyses.

**Results:**

The groups did not significantly differ in terms of female (*P* = 0.631) or male (*P* = 0.655) age. Sperm was obtained from 76/106 men in the m-TESE group (69.81%). The embryo transfer rate on day three was significantly higher in the m-TESE group (32.2% vs 8.3%; *P* = 0.003), whereas, on day five, it was significantly higher in the ejaculate group (61.7% vs 37.9%; *P* = 0.015). The ejaculate group had significantly higher overall pregnancy rates (59.1% vs 33%; *P* = 0.001) and overall live birth rates (37.9% vs 22.6%; *P* = 0.031).

**Conclusion:**

In recurrent ICSI failure, the reproductive success of ejaculate was higher than that of m-TESE. If it contains motile spermatozoa, the ejaculate before m-TESE could be considered even in severe OAT patients with two or more ART/ICSI failures.

Despite progress in assisted reproductive technologies (ART), couples experiencing severe male-factor infertility, specifically oligoasthenoteratozoospermia (OAT), still encounter considerable obstacles, particularly following recurrent unsuccessful intracytoplasmic sperm injection (ICSI) attempts. OAT is typically caused by congenital or acquired genitourinary tract anomalies and elevated reactive oxygen species levels. Nevertheless, the underlying cause remains unidentified in approximately 30%-50% of the cases (1). Male-factor infertility is defined as a decreased overall sperm concentration in the ejaculate (<15 million/mL), a decreased percentage of progressively motile sperm (<30%), and a diminished proportion of morphologically normal spermatozoa (<4%) across two separate seminal analyses (2).

A range of therapeutic options exists for individuals diagnosed with OAT. In patients who undergo two or more unsuccessful ART cycles, microdissection testicular sperm extraction (m-TESE) is proposed as a potential intervention (3). Although m-TESE currently represents the most effective surgical approach for sperm retrieval in severe OAT cases, its superiority over alternative techniques continues to be investigated (4). m-TESE is preferred over conventional TESE in patients with severe male-factor infertility and recurrent ART failures. As these patients often require repeated surgical procedures to retrieve viable sperm from the testicular tissue, m-TESE is used to minimize testicular tissue damage and the amount of tissue removed.

m-TESE is conducted under the assumption that testicular sperm may have higher genetic integrity or fertilization potential despite the invasive nature of the procedure (3,4). However, a considerable number of m-TESE patients still have occasional motile spermatozoa in their ejaculate, albeit in very low numbers. The question remains whether, in cases of severe OAT with prior ICSI failures, we should use ejaculated sperm if available or if m-TESE is unequivocally superior. The decision is often made on the day of oocyte retrieval, based on fresh semen analysis. No studies have directly compared these two pathways. Therefore, this retrospective study compared the reproductive outcomes in men with severe OAT and a history of ≥2 ART failures who underwent ICSI using sperm from ejaculate obtained on the morning of a planned m-TESE (leading to cancellation of the surgery) and those who proceeded with m-TESE for sperm retrieval.

## PARTICIPANTS AND METHODS

### Study population

We reviewed the medical records of 321 male infertility patients who underwent ICSI at the Department of Reproductive Endocrinology, Gülhane Training and Research Hospital between November 2016 and January 2024. The inclusion criteria were age 19-45 years, OAT diagnosis, and a history of at least two ART cycles without achieving pregnancy. OAT was confirmed by two semen analyses showing less than 1 million motile sperm, less than 5% progressive motility, and less than 1% normal sperm morphology (3). Several factors may account for previous unsuccessful ART cycles: complete fertilization failure, suboptimal embryo development, or unsuccessful embryo transfer procedures. Overall, 149 men were excluded due to other diagnosed causes of male infertility (including clinical varicocele – n = 24), discontinuation of ICSI for any reason (n = 19), or female infertility factors such as diminished ovarian reserve (n = 45), polycystic ovary syndrome (n = 27), secondary infertility (n = 21), or tubal factors (n = 13). Ultimately, 172 men were enrolled.

**Figure 1:**
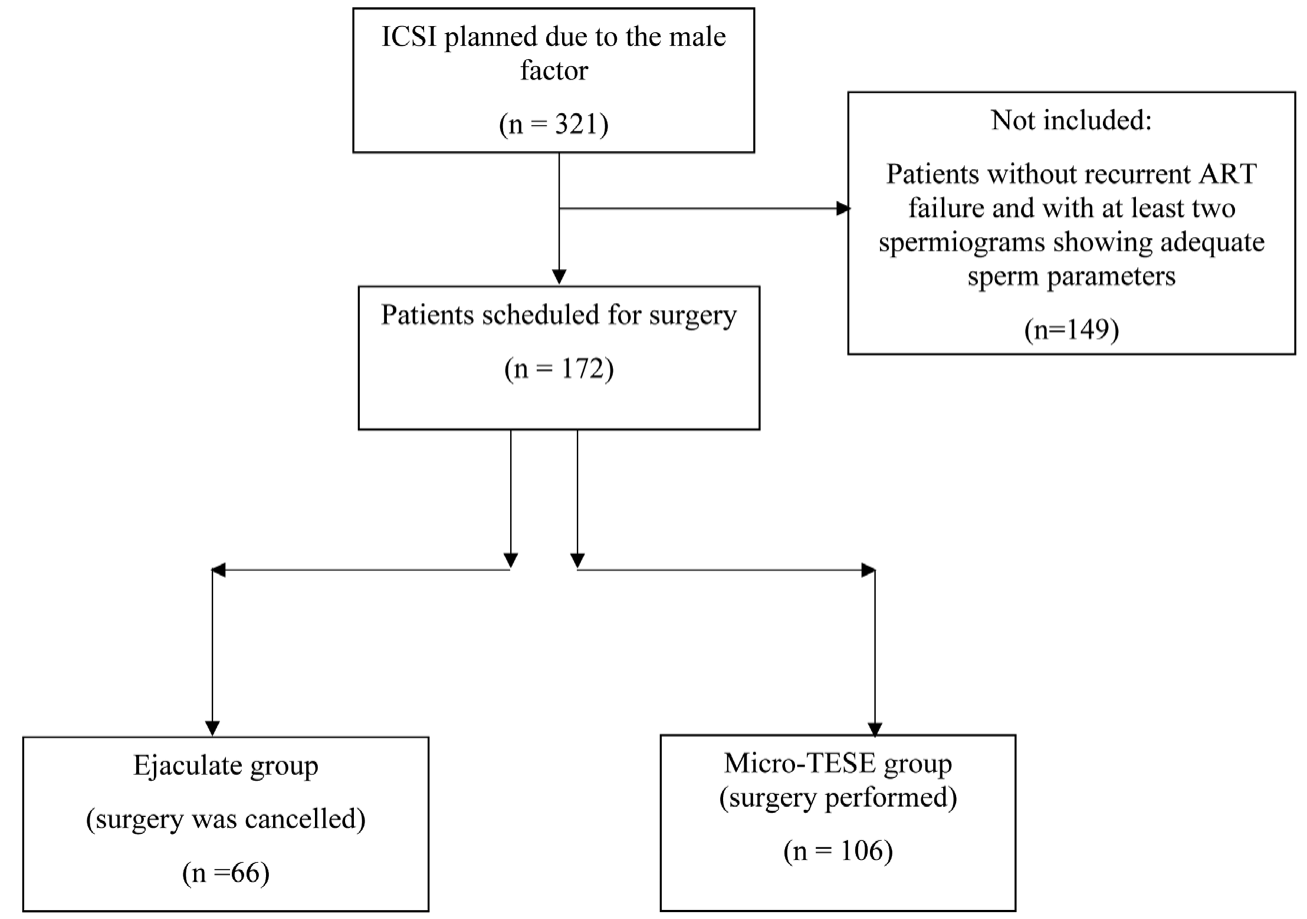
Study flowchart. ICSI – intracytoplasmic sperm injection; ART – assisted reproductive technologies.

Preoperative semen analysis identified motile sperm in 38.4% (66/172) of the men, leading to the cancellation of m-TESE on the morning of surgery. They were considered the ejaculate group, and 106 men were considered the m-TESE group (Figure 1).

All patients underwent medical history taking, physical examination, scrotal ultrasonography, hormonal profiling, and genetic analyses, including confirmation of normal karyotype, to elucidate underlying factors contributing to infertility.

### Ovarian stimulation protocol

Ovarian stimulation protocols for ICSI cycles were individualized according to the female partners’ clinical profiles (including age and body mass index [BMI]) and fertility criteria – ovarian reserve markers, antral follicle count (AFC), and concentrations of anti-Müllerian hormone (AMH), follicle-stimulating hormone (FSH), and luteinizing hormone (LH). Ovarian hyperstimulation was conducted using a cost-effective antagonist protocol known to yield higher live birth rates. Initially, gonadotropin preparations (recombinant FSH follicle-stimulating hormone or human menopausal gonadotropin) were administered, followed by gonadotropin-releasing hormone antagonists to prevent premature ovulation and ensure optimal timing for oocyte retrieval. The treatment was performed according to a flexible protocol, ie, when the leading follicles reached a mean diameter of 13-14 mm. When at least two follicles reached a mean diameter of 18 mm, recombinant human chorionic gonadotropin (hCG) was administered to promote final oocyte maturation and enhance pregnancy outcomes. Oocyte pickup (OPU) was performed 34-36 hours later, and hCG preparations were administered (5). Once the follicles matured, as defined by a dominant follicle size of 17-18 mm, oocytes were retrieved under anesthesia using transvaginal ultrasound guidance.

### m-TESE procedure

m-TESE was carried out according to Schlegel et al (6). All procedures were conducted in the urology operating suite under spinal or general anesthesia, with the patient positioned supine. The operation was collaboratively performed by two microsurgically trained urologists. A vertical incision was made to the scrotum, providing access to the testicular tissue. Subsequently, the tunica albuginea was exposed, and the testicular parenchyma was examined under surgical magnification to identify abnormally dilated seminiferous tubules (7). The most distinctly dilated tubules were selectively harvested as candidate sites for active spermatogenesis. Tissue samples were carefully excised with microsurgical instruments and promptly transferred to a HEPES-buffered medium dish. The specimens were immediately delivered to the laboratory for processing and evaluation (5,8). In patients with only immotile sperm in one testis, the opposite testis was examined. Bleeding was controlled with bipolar electrocautery. The tunica albuginea was then closed, and the testis was placed back in the scrotal area. The tunica vaginalis and skin tissue were closed anatomically (9).

### Intracytoplasmic sperm injection protocol

For all eligible participants meeting consistent criteria, ICSI procedures were conducted using freshly collected sperm obtained either through ejaculation or via m-TESE on the same day as oocyte retrieval. ICSI (10-12) represents a specialized assisted reproductive technique wherein one spermatozoon is injected directly into a metaphase II (MII) oocyte under microscopic guidance. Comparable success rates of ICSI have been documented using spermatozoa derived from ejaculate or testicular extraction (13,14).

### Evaluation of embryo quality

A cleavage-stage embryo is considered of high quality when it reaches four to five blastomeres with less than 20% fragmentation by day 2 of culture or develops at least eight blastomeres with similarly low fragmentation by day 3. These embryos exhibit uniformly sized blastomeres and an absence of multinucleation. Indicators of poor embryo quality include accelerated or delayed cleavage, fragmentation exceeding 10%, uneven blastomere size, an enlarged perivitelline space, and cytoplasmic irregularities. Embryo quality is considered notably declined when fragmentation surpasses 25% or when multinucleated blastomeres are present (15). Mature oocyte number is an important determinant for ART success. Since all couples with female infertility factors were excluded from the study, the number of mature oocytes was specifically reported to emphasize that it did not negatively affect pregnancy outcomes.

### Embryo transfer

Following retrieval, oocytes were categorized according to nuclear maturation status. Those identified as mature were microinjected with sperm obtained via m-TESE or ejaculation. Successfully fertilized oocytes were then cultured under controlled conditions and monitored for developmental progression. Embryo transfer was performed on day 3, 4, or 5 postfertilization, based on morphological development, using a soft catheter and visualization with transabdominal ultrasound. This procedure may be implemented within fresh embryo transfer cycles (16). We prefer blast embryo transfer, but in patients with challenging clinical profiles, embryo transfer was performed on day 3 for embryos that could not be cultured until day 5 or had no chance to be chosen due to a limited number of embryos. Clinical pregnancy was confirmed by the detection of a fetal heartbeat within the intrauterine gestational sac via ultrasound.

### Ethical considerations

The study was approved by the Ethics Committee of Gülhane Training and Research Hospital. All treated patients routinely provided signed informed consent, in accordance with the Declaration of Helsinki.

### Statistical analysis

The sample size was determined with G*Power, version 3.1.9.4 (Franz Faul, Universität Kiel, Germany). Calculation assumptions included a projected effect size of 0.71, an alpha error of 5%, and statistical power of 95%. A minimum of 53 participants per group was required to achieve adequate study power (9).

The normality of data distribution was evaluated with the Kolmogorov-Smirnov test. Continuous variables are reported as mean ± standard deviation or as median with minimum and maximum values, while categorical variables are expressed as counts and percentages. Differences in continuous variables between the groups were evaluated with the independent-samples *t* test, the Mann-Whitney U test for two-group comparisons, or the Kruskal-Wallis test for multiple-group comparisons. Differences in categorical variables were evaluated using the χ^2^ tests or Fisher exact test. As the generalized estimating equations analysis yielded a *P* value of 0.106, no subgroup analysis was conducted. Multivariate logistic regression analysis was performed to identify independent risk factors for undergoing m-TESE. The significance level was set at *P* < 0.05. Statistical analysis was conducted with IBM SPSS, version 26.0 (IBM Corp., Armonk, NY, USA).

## RESULTS

Of the 172 patients with OAT scheduled for m-TESE, 66 (38.4%) were in the ejaculate group and 106 patients were in the m-TESE group. Sperm was obtained in 71.7% (76/106 patients). No significant differences were found between the groups in terms of age or BMI for either women (30 [20-42] vs 31 [21-43] years, *P* = 0.808; 26.86 ± 3.12 kg/m^2^ vs 26.21 ± 3.24 kg/m^2^ *P* = 0.199) or men (32 [23-41] vs 33 [22-41] years, *P* = 0.923; 29.15 ± 1.75 kg/m^2^ vs 29.36 ± 1.48 kg/m^2^, *P* = 0.406). Additionally, no significant differences were found in the female endocrine profile, male endocrine profile (FSH, LH, and testosterone), or genetic analysis. The m-TESE group demonstrated a significantly lower total motile sperm count than the ejaculate group (*P* = 0.001). As anticipated, the m-TESE group had a substantially higher prevalence of azoospermia during m-TESE (28.3%, 30/106 patients) than the ejaculate group (0%).

Out of the 106 patients who underwent m-TESE, 42 (39.6%) required bilateral m-TESE. Sperm retrieval during m-TESE was unsuccessful for 30/42 (71.4%) patients, but their post-operative pathologies revealed normal histopathological results (Table 1).

**Table 1:** Demographic characteristics in the ejaculate and microdissection testicular sperm extraction (m-TESE) groups*

Characteristics	Ejaculate (n = 66)	m-TESE (n = 106)	P value
**Female factor**			
Age (years)	30 (20-42)	31 (21-43)	0.808^†^
Body mass index, kg/m^2^	26.86 ± 3.12	26.21 ± 3.24	0.199^‡^
Anti-Müllerian hormone, ng/mL	1.91 ± 0.95	2.31 ± 1.67	0.077^‡^
Follicle stimulant hormone, mIU/mL	7.65 ± 1.77	7.71 ± 2.68	0.882^‡^
Luteinizing hormone, mIU/mL	5.14 ± 3.19	4.21 ± 1.41	0.494^‡^
Antral follicle count	8.44 ± 3.31	7.78 ± 3.77	0.240^‡^
**Male factor**			
Age (years)	32 (23-41)	33 (22-41)	0.923^†^
Body mass index, kg/m^2^	29.15 ± 1.75	29.36 ± 1.48	0.406^‡^
Total motile sperm concentration, 10^6^/mL	0.23 ± 0.49	0.03 ± 0.12	**0.001** ^‡^
Follicle stimulant hormone, mIU/mL	5.53 ± 1.25	5.87 ± 1.86	0.861^‡^
Luteinizing hormone, mIU/mL	6.14 ± 2.28	5.81 ± 1.45	0.445^‡^
Testosterone, ng/dL	325.2 ± 1.23	337 ± 1.36	0.654^‡^
Bilateral m-TESE (42 of 106)	0 (0)	42/106 (39.6)	
Absence of sperm in bilateral m-TESE	0 (0)	30/42 (71.4)	
Positive sperm in bilateral m-TESE		12/42 (28.6)	

*Data are presented as median (min-max) or mean ± standard deviation or number (%).

†Mann-Whitney U test.

‡t test.

The m-TESE group had undergone a significantly higher number of ICSI cycles than the ejaculate group (1.79 vs 2.21 cycles, *P* = 0.046). In terms of ovarian response, the m-TESE group exhibited a higher number of oocytes retrieved per cycle, but the difference was not significant (9.86 vs 12.36, *P* = 0.054). The number of mature oocytes (MII) was significantly greater in the first cycle (7.59 vs 9.82, *P* = 0.045). Cumulative analysis across all cycles further demonstrated a significantly reduced total oocyte yield in the ejaculate group (7.98 vs 10.41, *P* = 0.030). The groups did not significantly differ in the number of fertilized oocytes (two pronuclei) or the number of embryo transfers in the first cycle. The m-TESE group had a significantly higher rate of embryo transfer on day 3 (32.2% vs 8.3%, *P* = 0.003), whereas the ejaculate group had a higher rate of embryo transfer on day 5 (61.7% vs 37.9%, *P* = 0.015) (Table 2).

**Table 2:** Intracytoplasmic sperm injection cycle and pregnancy outcomes in the ejaculate and microdissection testicular sperm extraction (m-TESE) groups*

Cycles		Ejaculate group (n = 66)		m-TESE group (n = 106)		*P* Value
Cycle number	1.79 ± 1.19		2.21 ± 1.26		0.046*	
**First cycle outcomes**						
oocyte retrieved	9.86 ± 5.58		12.36 ± 9.39		0.054*	
metaphase II oocyte retrieved	7.59 ± 5.02		9.82 ± 7.49		0.045*	
two pronuclei	5.42 ± 3.62		4.78 ± 3.84		0.331*	
embryo transfer (ET)	1.31 ± 0.55		1.53 ± 1.27		0.246*	
total fertilization failure	4 (6.1)		2 (1.9)		0.205^†^	
poor embryo quality	2 (3.0)		9 (8.5)		0.208^†^	
day 3 of ET	4 (8.3)		19 (32.2)		**0.003** ^‡^	
day 4 of ET	15 (31.3)		18 (31.0)		0.981^‡^	
day 5 of ET	29 (61.7)		22 (37.9)		**0.015** ^‡^	
**Pregnancy outcome**						
pregnancy	30 (45.5)		27 (25.5)		**0.007** ^‡^	
live birth	22 (33.3)		23 (21.7)		0.091^‡^	
multiple pregnancy	0 (0)		4 (14.8)		1.480^‡^	
abortion	8(26.6)		4(14.8)			
term delivery	22 (73.3)		23 (85.2)			
cesarean delivery rate	20 (90.9)		20 (86.9)		0.667^‡^	

*t-test.

†Fisher exact test was used in cases of the minimum expected count <2 or the expected count <5 in more than 20% of the cells.

‡χ^2^ test.

**Figure 2:**
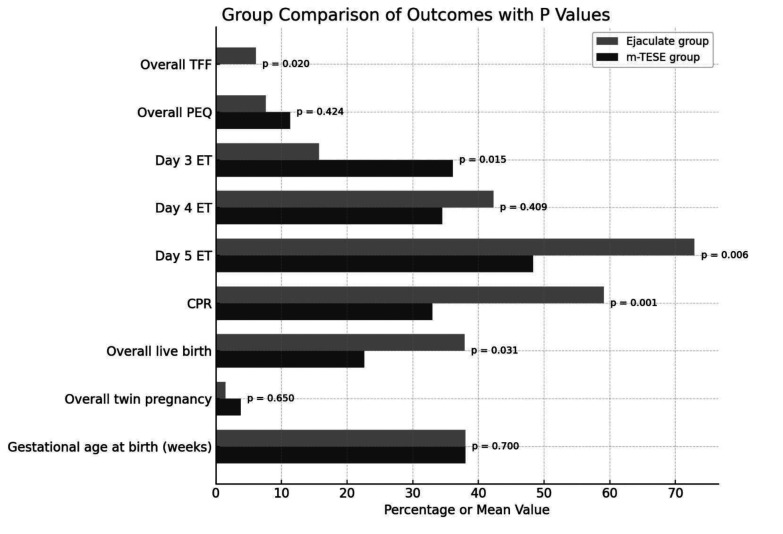
Outcomes of the overall cycles. ET – embryo transfer; PEQ – poor embryo quality; TFF – total fertilization failure; PN – pronuclei; MII – metaphase II oocyte; CPR – clinical pregnancy rate. *Fisher exact test.

The groups did not differ in terms of total fertilization failure, poor embryo quality, number of embryo transfers, gestational age at delivery, or infant birth weight. However, the ejaculate group demonstrated significantly higher rates of overall pregnancy (59.1% vs 33.0%, *P* = 0.001) and live births (37.9% vs 22.6%, *P* = 0.031) (Figure 2).

There was no difference in the number of mature oocytes according to embryo transfer days; however, as expected, the number of two pronuclei was significantly higher on day 3 (Table 3).

**Table 3:** The number of metaphase II (MII) oocytes and two pronuclei (2PN) according to embryo transfer day*

	Embryo transfer			
	Day 3	Day 4	Day 5	P value^‡§^
2PN	3 (1-12)	5 (1-13)	6 (1-19)	**0.005** ^†^
MII oocyte	6 (1-41)	7.5 (3-20)	9 (1-30)	0.081
Total oocyte retrieval	8 (2-54)	11.5 (3-24)	10 (3-42)	**0.043** ^‡^

*Data are presented as median (interquartile range).

†day 3 vs day 5 (*P* = 0.001); day 3 vs day 4 (*P* = 0.019); day 4 vs day 5 (*P* = 0.456).

‡day 3 vs day 5 (*P* = 0.023); day 3 vs day 4 (*P* = 0.024); day 4 vs day 5 (*P* = 0.844).

§Kruskal-Wallis test was used for three-group comparisons, and the Mann-Whitney U test was used for two-group comparisons.

Multivariate logistic regression analysis showed that independent risk factors for undergoing m-TESE were two pronuclei (OR 0.773, 95% CI 0.636-0.940) and overall mature oocyte count (OR 1.249, 95% CI 1.087-1.435) (Table 4).

**Table 4:** Multivariate logistic regression analysis assessing independent risk factors for undergoing microdissection testicular sperm extraction

	Odds ratio Exp (B)	95% confidence interval
Age (female)	0.951	0.694-1.302
Age (male)	1.089	0.753-1.577
Overall oocyte	0.961	0.802-1.151
Overall mature oocyte	1.249	1.087-1.435
Overall two pronuclei	0.773	0.636-0.940
Overall embryo transfer	0.747	0.498-1.120

## DISCUSSION

In this study, compared with the m-TESE group, the ejaculate group achieved significantly higher overall pregnancy and live birth rates. Earlier research demonstrated comparable gestational ages and newborn birth weights between m-TESE, TESE, and ejaculated sperm in ICSI cycles. Another study found no significant differences in the mean number of oocytes retrieved and mature oocytes, fertilization rates, or the proportion of high-quality embryos between synchronous ICSI (using fresh sperm on the day of oocyte retrieval) vs asynchronous ICSI (using cryopreserved-thawed sperm). The two approaches also did not significantly differ in terms of clinical pregnancy rates, live birth rates, and the ratio of fresh to frozen-thawed embryo transfers. However, the asynchronous ICSI group had a higher incidence of pregnancy loss (9). In the current study, all ICSI procedures were performed simultaneously with oocyte retrieval. The ejaculate group had a higher number of good-quality embryos (day-5 blastocysts), clinical pregnancy rate, live birth rate, and the number of abortions (although not statistically significant), and 2 pronuclei. The m-TESE group exhibited significantly more mature oocytes, as we decided to retrieve a larger number of oocytes in patients undergoing m-TESE given the potential need for repeated surgical procedures. Conversely, the higher rates of 2 pronuclei and improved reproductive outcomes in the ejaculate group can be attributed to the superior quality of sperm obtained through ejaculation. The significantly higher clinical pregnancy and live birth rates in the ejaculate group, despite lower oocyte yields, underscore the importance of the functional maturity of sperm over retrieval success. This divergence may be rooted in the fundamental biological differences between ejaculated and testicular sperm. While ICSI mechanically bypasses natural barriers to fertilization, it cannot fully compensate for deficiencies in sperm maturation (12). Ejaculated sperm have completed their transit through the epididymis, a process critical for acquiring motility and fertilization competence. More importantly, post-testicular maturation is vital for the complete epigenetic programming of sperm DNA, and thus proper embryonic genome activation and development (13). The superior outcomes achieved with ejaculated sperm likely reflect its more favorable epigenetic and functional profile. This speculation is supported by studies showing comparable results between ejaculated and epididymal sperm (13), and lower fertilization potential in immotile testicular sperm (14). These findings emphasize that the source and maturity of sperm determine ICSI success beyond the mere presence of spermatozoa. A previous study among patients with severe oligospermia (17) found markedly higher pregnancy rate with sperm derived from fresh testicular tissue vs ejaculate. Another study examining m-TESE ICSI outcomes in severe oligospermia patients reported no sperm retrieval in 59.5% of patients during initial microdissection procedures. Among the remaining couples, clinical pregnancy and live birth rates reached 21.7% and 20.6%, respectively (18). In our investigation, sperm retrieval failed in 28.3% of m-TESE patients. Among those with successful retrieval, live birth and overall pregnancy rates were 22.6% and 33%, respectively. Reproductive outcomes were consistently more favorable in the ejaculate group than in the m-TESE group. Another study evaluating ICSI outcomes using fresh vs cryopreserved m-TESE-derived sperm in severe oligospermia patients reported a 35.25% sperm retrieval rate. The groups did not differ in demographics, fertilization rates, or embryo quality, but the cryopreserved group had a significantly higher miscarriage and a lower birth rate (8). In our cohort, the ejaculate group achieved higher obstetric success compared with the m-TESE group. Notably, all cycles were conducted with fresh sperm.

Numerous comparative studies have demonstrated superior sperm retrieval rates of m-TESE over conventional techniques (19-22), particularly in cases involving Y-chromosome microdeletions (23). Another study of reproductive outcomes and surgical timing (7) reported a mean patient age of 33.3 years and a sperm retrieval rate of 55.4%. Fifteen of 16 clinical pregnancies resulted in a live birth. The factors predicting sperm retrieval success included duration of marriage and a history of infertility. Live birth rates were highest in men under 35.2 years of age with severe oligospermia and female partners under 36.9 years of age (7). The mean age in our series was 32.33 ± 4.30 years. Within this cohort, the cumulative pregnancy rate reached 33%, while the live birth rate was 22.6%.

A recent investigation examining the timing of micro-TESE procedures reported moderately improved sperm retrieval rates and fertilization rates when surgical sperm retrieval was performed on the same day as oocyte pickup (24). However, no significant differences were observed in clinical pregnancy rates or live birth rates between same-day and previous-day procedures. The authors concluded that the timing of micro-TESE relative to oocyte pickup does not significantly influence clinical outcomes (24). In our investigation, all m-TESE procedures were conducted on the same day as OPU.

A potential limitation of our study stems from its retrospective design, as some patients were excluded due to incomplete documentation of certain clinical parameters. Another limitation was its single-center design, limiting the generalizability of our findings. Despite these limitations, this study showed that ejaculated sperm can be a good treatment option, even for patients with OAT who are scheduled for m-TESE and have had two or more failed ART attempts. Therefore, counseling couples and recommending ICSI with pre-m-TESE ejaculated sperm could be a viable option.
